# Dr. C. W. Spalding

**Published:** 1896-10

**Authors:** H. J. McKellops, Wm. N. Morrison, J. B. Newby

**Affiliations:** Committee of St. Louis Dental Society. St. Louis, Mo.; Committee of St. Louis Dental Society. St. Louis, Mo.; Committee of St. Louis Dental Society. St. Louis, Mo.


### Obituary.

### DR. C. W. SPALDING.

Died,—At Riverpoint, R. I., June 9th, 1896, Dr. C. W. Spalding, in the 82nd year of his age.

Dr. Christopher W. Spalding was born March 15th, 1814, in Rhode Island, of Scotch origin. His ancestors were prominent in the Revolutionary war.

Dr. Spalding received a common school education. In the same year, he began his professional studies, and received the degree of Doctor of Dental Surgery in 1851, and that of Doctor of Medicine in 1869.

In 1847-48, Dr. Spalding spent a year in savannah, Ga. In 1849, he removed from Ithaca, N. Y., to St. Louis, where he resided for many years.

He took a leading part in the organization of the Western Dental Society in 1851, and has been prominently identified with the American, several State and City associations, and has been actively engaged in every good work which would tend to elevate the profession of his choice.

He was also an ardent advocate of the Homœopathic school of remedies, and was very successful in their administration.

In 1838, Dr. Spalding married Miss Cornelia Anna Erb, also of revolutionary stock. Of this union there is one son, Dr. John H. Spalding, who succeeded to his father's practice in this city.

Dr. Spalding and wife, whose death preceded his over a year and a half, removed to Rhode Island, August, 1890, to spend their declining years in the State of their nativity.

In the death of Dr. Spalding, we have lost an earnest and faithful laborer in the healing art, and

*Resolved*, That a memorial page be set aside in the Society's Transactions and a copy furnished the journals for pub

H. J. McKELLOPS,
WM. N. MORRISON.
J. B. NEWBY,
Committee of St. Louis Dental Society.

ST. LOUIS, MO., June 19th, 1896.

